# Isolation of Cellulose-Degrading Bacteria and Determination of Their Cellulolytic Potential

**DOI:** 10.1155/2012/578925

**Published:** 2012-01-18

**Authors:** Pratima Gupta, Kalpana Samant, Avinash Sahu

**Affiliations:** ^1^Department of Biotechnology, National Institute of Technology, Raipur 492 010, India; ^2^Indian Institute of Technology Delhi, New Delhi 110016, India

## Abstract

Eight isolates of cellulose-degrading bacteria (CDB) were isolated from four different invertebrates (termite, snail, caterpillar, and bookworm) by enriching the basal culture medium with filter paper as substrate for cellulose degradation. To indicate the cellulase activity of the organisms, diameter of clear zone around the colony and hydrolytic value on cellulose Congo Red agar media were measured. CDB 8 and CDB 10 exhibited the maximum zone of clearance around the colony with diameter of 45 and 50 mm and with the hydrolytic value of 9 and 9.8, respectively. The enzyme assays for two enzymes, filter paper cellulase (FPC), and cellulase (endoglucanase), were examined by methods recommended by the International Union of Pure and Applied Chemistry (IUPAC). The extracellular cellulase activities ranged from 0.012 to 0.196 IU/mL for FPC and 0.162 to 0.400 IU/mL for endoglucanase assay. All the cultures were also further tested for their capacity to degrade filter paper by gravimetric method. The maximum filter paper degradation percentage was estimated to be 65.7 for CDB 8. Selected bacterial isolates CDB 2, 7, 8, and 10 were co-cultured with *Saccharomyces cerevisiae* for simultaneous saccharification and fermentation. Ethanol production was positively tested after five days of incubation with acidified potassium dichromate.

## 1. Introduction

Cellulose is a linear polysaccharide of glucose residues with *β*-1, 4-glycosidic linkages. Abundant availability of cellulose makes it an attractive raw material for producing many industrially important commodity products. Sadly, much of the cellulosic waste is often disposed of by biomass burning, which is not restricted to developing countries alone, but is considered a global phenomenon. With the help of cellulolytic system, cellulose can be converted to glucose which is a multiutility product, in a much cheaper and biologically favourable process.

Cellulolysis is basically the biological process controlled and processed by the enzymes of cellulase system. Cellulase enzyme system comprises three classes of soluble extracellular enzymes: 1, 4-*β*-endoglucanase, 1, 4-*β*-exoglucanase, and *β*-glucosidase (*β*-D-glucoside glucohydrolase or cellobiase). Endoglucanase is responsible for random cleavage of *β*-1, 4-glycosidic bonds along a cellulose chain. Exoglucanase is necessary for cleavage of the nonreducing end of a cellulose chain and splitting of the elementary fibrils from the crystalline cellulose, and *β*-1, 4-glucosidase hydrolyses cellobiose and water-soluble cellodextrin to glucose [1, 2]. Only the synergy of the above three enzymes makes the complete cellulose hydrolysis to glucose [3–5] or a thorough mineralization to H_2_O and CO_2_ possible.

Source for cellulase system extraction is best suitable from microbial system found in the gut of organisms thriving on cellulosic biomasses as their major feed. Insects like termites (*Isopteran*), bookworm (*Lepidoptera*), and so forth, are found to have syntrophic symbiotic microflora in their guts responsible for cellulosic feed digestion [6, 7]. Many microorganisms have been reported with cellulosic activities including many bacterial and fungal strains both aerobic and anaerobic. *Chaetomium, Fusarium Myrothecium, Trichoderma. Penicillium, Aspergillus, and so forth, *are some of the reported fungal species responsible for cellulosic biomass hydrolysation. Cellulolytic bacterial species include *Trichonympha, Clostridium, Actinomycetes, Bacteroides succinogenes, Butyrivibrio fibrisolvens, Ruminococcus albus, and Methanobrevibacter ruminantium *[8, 9].

Cellulase due to its massive applicability has been used in various industrial processes such as biofuels like bioethanol [10, 11], triphasic biomethanation [12]; agricultural and plant waste management [13, 14]; chiral separation and ligand binding studies [15].

The present work concentrates on the isolation of cellulose-degrading bacteria from invertebrates such as termites, snails, caterpillars, and bookworms and assessment of their cellulolytic activity. The coculturing of cellulose-degrading bacteria and yeast was also carried out for simultaneous saccharification and fermentation of cellulose into ethanol.

## 2. Materials and Methods

### 2.1. Sample Collection

Cellulose feeding organisms like termite, caterpillar, bookworm, and snail were collected for isolation of cellulose-degrading bacteria from woody habitats. Guts of the collected organism were separately crushed in 0.9% saline solution under sterile condition.

### 2.2. Isolation and Screening of Cellulose-Degrading Bacteria

The macerated gut of the collected organisms was inoculated in a basal salt media (NaNO_3_ 2.5 g; KH_2_PO_4_ 2 g; MgSO_4_ 0.2 g; NaCl 0.2 g; CaCl_2_·6H_2_O 0.1 g in a liter) containing filter paper (Whatman filter paper no. 1 of area 70.541 cm^2^) for the isolation of cellulolytic bacteria. These cultures were incubated for 7 days in a shaker incubator at 37°C at 100 rpm. Bacterial colonies capable of utilizing cellulose as sole source of carbon were isolated on cellulose agar media composed of KH_2_PO_4_ 0.5 g MgSO_4_ 0.25 g cellulose 2.0 g agar 15 g gelatin 2 g and distilled water lL and at pH 6.8–7.2.

Confirmation of cellulose-degrading ability of bacterial isolates was performed by streaking on the cellulose Congo-Red agar media with the following composition: KH_2_PO_4_ 0.5 g, MgSO_4_ 0.25 g, cellulose 2 g, agar 15 g, Congo-Red 0.2 g, and gelatin 2 g; distilled water 1 L and at pH 6.8–7.2. The use of Congo-Red as an indicator for cellulose degradation in an agar medium provides the basis for a rapid and sensitive screening test for cellulolytic bacteria. Colonies showing discoloration of Congo-Red were taken as positive cellulose-degrading bacterial colonies [13], and only these were taken for further study. Cellulose-degrading potential of the positive isolates was also qualitatively estimated by calculating hydrolysis capacity (HC), that is, the ratio of diameter of clearing zone and colony [16].

### 2.3. Enzyme Production

The selected CDB isolates were cultured at 37°C at 150 rpm in an enzyme production media composed of KH_2_PO_4_ 0.5 g, MgSO_4_ 0.25 g, and gelatin 2 g, distilled water 1 L and containing Whatman filter paper No.1 (1 × 6 cm strip, 0.05 g per 20 mL) and at pH 6.8–7.2. Broth culture after three days of incubation period was subjected to centrifugation at 5000 rpm for 15 min at 4°C. Supernatant was collected and stored as crude enzyme preparation at 4°C for further enzyme assays. Pellet recovered after centrifugation of broth culture was subjected to gravimetric analysis in order to determine the residual cellulose of filter paper [17].

### 2.4. Enzyme Assay

Total cellulose activity was determined by measuring the amount of reducing sugar formed from filter paper. Endoglucanase (*β*1-4 endoglucanase-EC 3.2.1.4) activity was assayed by measuring the amount of reducing sugar from amorphous cellulose. The enzyme activity was determined according to the methods recommended by the International Union of Pure and Applied Chemistry (IUPAC) commission on biotechnology [18]. Endoglucanase activity was determined by incubating 0.5 mL of supernatant with 0.5 mL of 2% amorphous cellulose in 0.05 m sodium citrate buffer (pH 4.8) at 50 for 30 min. FPC activity was determined by incubating 0.5 mL of supernatant with 1.0 mL of 0.05 M sodium citrate buffer (pH4.8) containing Whatman no.1 filter paper strip—1.0 × 6.0 cm (=50 mg). After incubation for an hour at 50°C, the reaction was terminated by adding 3 mL of 3, 5-dinitrosalicylic acid (DNS) reagent to 1 mL of reaction mixture. In these tests, reducing sugars were estimated spectrophotometrically with 3, 5-dinitrosalicylic acid [19] using glucose as standards. The enzymatic activity of total FPCase and endoglucanase were defined in international units (IU). One unit of enzymatic activity is defined as the amount of enzyme that releases 1 *μ*mol reducing sugars (measured as glucose) per mL per minute.

### 2.5. Bioethanol Production

A total of four isolates CDB 2, 7, 8, and 10 were grown in mixed culture using basal salt medium in two different sets, one containing filter paper and the other containing cellulose powder as substrate for production of cellulolytic enzyme and to initiate saccharification process. Culture was incubated at 37°C with mixing at 100 rpm for 3 days. After completion of three days of incubation, the above culture broth was conditioned for coculturing of *Saccharomyces cerevisae *by addition of filter-sterilized salt solution (KH_2_PO_4_ 0.4 g, MgSO_4_ 0.02 g, CaCO_3_ 0.05 g, and NaCl 0.01 g to 1 L culture broth). The simultaneous saccharification and fermentation was carried out at 27°C for 5 days in stationary condition. At the end of incubation, the culture broth was qualitatively tested for alcohol production using the K2Cr2O7 reagent test [20].

## 3. Result and Discussion

### 3.1. Isolation and Screening of Cellulose-Degrading Bacteria

**Figure 1:**
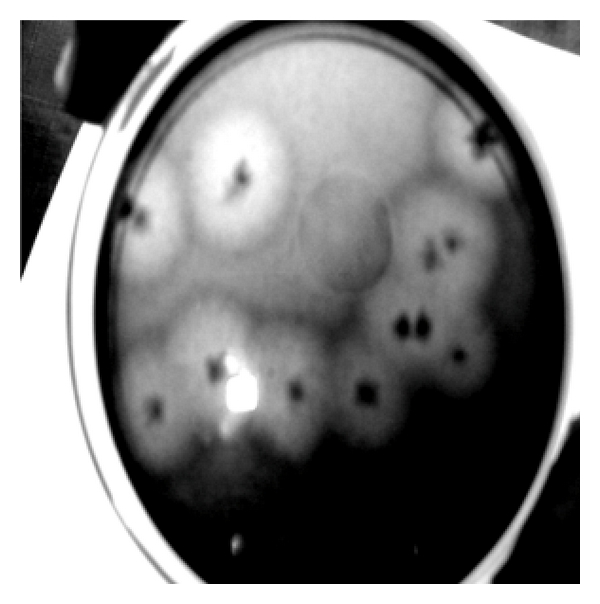
Zone of clearance on cellulose Congo Red agar plates for isolate CDB 10 after 48 hrs of incubation. The formation of clearing zone around the colonies confirms the secretion of extracellular cellulase.

Cellulose degrading bacteria were enriched and isolated by inoculating filter paper in liquid medium with macerated guts from termite, bookworm, snail, and caterpillar separately. All bacterial culture showed growth as the medium turned cloudy and the filter paper became macerated. Cellulolytic bacteria were also isolated from gut of insects by R. J. Dillon and V. M. Dillon. [6], Wenzel et al. [21], Delalibera et al. [22], and Ramrn et al. [23]. A total of eight bacterial isolates found to be positive on screening media (cellulose Congo-Red agar) producing clear zone (as shown in Figure 1) during aerobic incubation were as follows: CDB 1, 2, 8, and 9 from termite, CDB 6 and 10 from snail, CDB 3 from bookworm, and CDB 7 from caterpillar. The result showed that clearing zone and HC value ranged to bebetween 28.0 to 50.0 mm and 4.3 to 9.0 for all isolates (Table 1). The range of HC value obtained is similar to range reported by Lu et al. [24] whereas Hatami et al. [25] found the hydrolytic value between 1.38 to 2.33 and 0.15 to 1.37 of cellulolytic aerobic bacterial isolates from farming and forest soil, respectively.

### 3.2. Cellulolytic Potential of Bacterial Isolates

**Figure 2:**
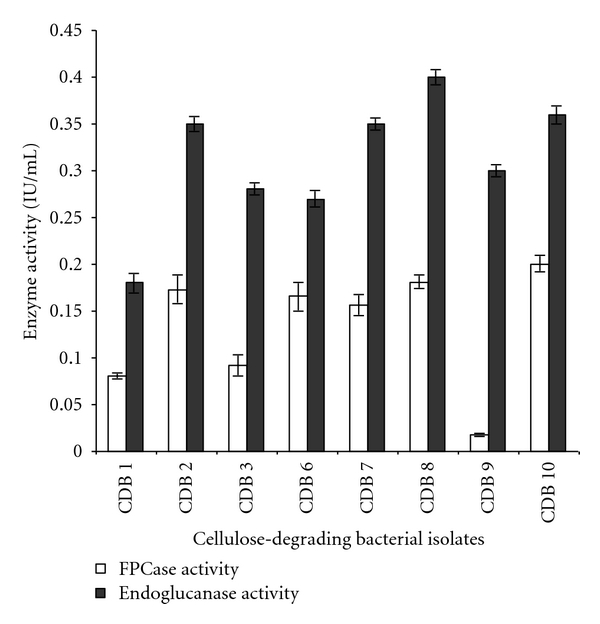
Extracellular cellulase activity of two enzymes (FPCase and endoglucanase) of all CDBs isolates. The activities ranged from 0.012 to 0.196 IU/mL for FPCase and 0.1622 to 0.400 IU/mL for endoglucanase assay. Values in figure are means of three replicates with standard deviation.

**Figure 3:**
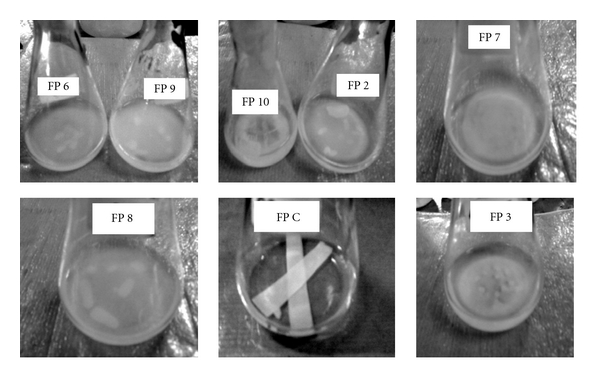
Filter paper degradation by isolates CDB 2, 3, 6, 7, 8, 9, and 10 cultured in basal salt medium supplemented with Whatman filter paper no.1 (1 × 6 cm strip × 2, 0.05 g per 20 mL) at the end of 96 hrs of incubation. Flask FP C is the control for this experimental set up and does not show any filter paper degradation.

**Figure 4:**
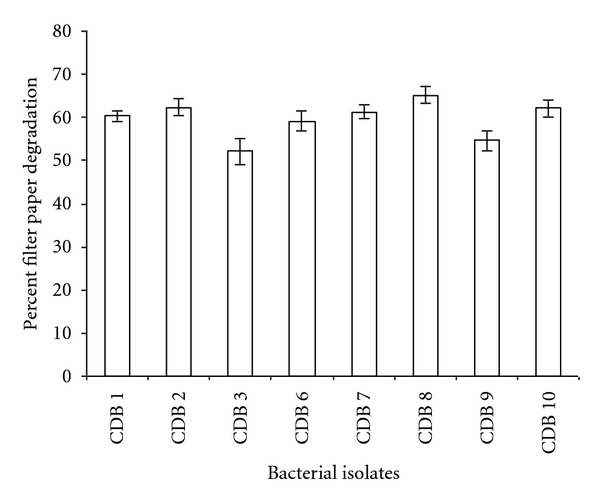
Percent filter paper degradation by various bacterial isolates obtained from termite, snail, bookworm, and caterpillar by gravimetric method. Maximum percentage of filter paper degradation was found to be 65.7% by CDB 8. Values in figure are means of three replicates with standard deviation.

A total of eight positive isolates (CDB1, 2, 3, 6, 7, 8, 9, and 10) were selected for enzyme production and their respective cellulolytic activity was estimated. Enzyme assay for cellulase activity on filter paper was found to be highest for CDB 10 with 0.194 IU/mL, while for endoglucanase assay maximum activity was determined to be 0.400 IU/mL by CDB 8. The activities ranged from 0.012 to 0.196 IU/mL for FPCase and 0.1622 to 0.400 IU/mL for endoglucanase assay. The two isolates CDB8 and CDB10 exhibited the highest extracellular cellulase activities compared to other isolates as shown in activity assay performed for all isolates in Figure 2. Similar results were reported for *Acinetobacter anitratus* and *Branhamella* sp. grown in a basic salt medium with glucose and CMC as sole carbon source separately. Ekperigin [10] quantitatively determined the cellulase degrading enzyme of *A. anitratus* and *Branhamella* sp. The maximum enzyme activities of *A. anitratus* culture supernatant were 0.48 and 0.24 U/mL for CMC and glucose, respectively. For *Branhamella *sp., the maximum enzyme activities of the culture supernatant were 2.56 and 0.34 U/mL for CMC and glucose, respectively. The filter paper degradation was observed separately in CDB 2, 3, 6, 7, 8, 9, and 10 as shown in Figure 3. Gravimetric analysis shows that maximum and minimum rates of filter paper degradation were 65.7% and 55%, respectively, estimated at third day of incubation. An average of 57.64% degradation rate was computed.  Figure 4 shows that CDB 8 has highest filter paper degradation rate of 65.7%. In a result documented by Lu et al. [13], the data for synergetic cellulose degradation detected in four groups of mixed cultures were only 23.5%, 26.3%, 19.4%, and 24.5%, respectively. Bichet-Hebe et al. [26] reported the rates of paper degradation ranged from 31 to 60% after 10 days for mixed bacterial populations by gravimetric procedure. 

### 3.3. Bioethanol Production

The experiment setup for simultaneous saccharification and fermentation of mixed bacterial culture (CDB, 2, 7, 8, and 10) with *Saccharomyces cerevisiae* resulted in production of ethanol. This result expressed the high cellulolytic potential of these selected bacterial isolates for decomposition of cellulose and its fermentation for production of ethanol. Satheesh Kumar et al. [27] also used Whatman filter paper and cellulose powder as substrate in submerged fermentation for production of cellulolytic enzymes by *Bacillus* sp. FME (flour mill effluent). Coculturing of bacterial strains with yeast sp. and simultaneous saccharification and fermentation of ethanol were reported by several workers (Lenziou et al. [28] and Eklund and Zacchi [29]). Results indicated that significant synergistic cellulose degradation can be achieved in mixed culture system of cellulolytic bacteria and noncellulolytic yeast in which noncellulolytic yeast, *Saccharomyces cerevisiae* utilizes the reducing sugar derived from cellulose degradation and converts it to ethanol.

The bacterial isolates showed a potential to convert cellulose into reducing sugars which could be readily used in many applications like feed stock for production of valuable organic compounds; for example in the present study this has been demonstrated by simultaneous saccharification and fermentation of cellulose into ethanol.

## Figures and Tables

**Table 1:** Maximum clearing zone and hydrolytic capacity (HC) value of CDB on cellulose Congo red agar media. This table shows the assessment of bacterial isolates from the different source organism for cellulose decomposition via measurement of clear zone around the colony and calculation of hydrolytic value in cellulose Congo Red media. Maximum clearing zone of 50 mm and HC value of 9.8 were estimated for CDB 10.

Source organism	Isolate number	Maximum clearing zone (mm)	Average hc value	Maximum HC value
Termite	CDB1	30	5.49	6.77
	CDB2	42	4.29	8.4
	CDB8	45	5.36	9
	CDB9	28	4.32	4.39

Snail	CDB6	40	3.45	6.45
	CDB10	50	5.96	9.8

Bookworm	CDB3	30	3.51	4.3

Caterpillar	CDB7	50	5.35	8.2
